# 5,7-Dimethoxycoumarin ameliorates vincristine induced neuropathic pain: potential role of 5HT_3_ receptors and monoamines

**DOI:** 10.3389/fphar.2023.1213763

**Published:** 2023-10-17

**Authors:** Muhammad Usman, Hurmat Malik, Ahmed Tokhi, Mehreen Arif, Zilli Huma, Khalid Rauf, Robert D. E. Sewell

**Affiliations:** ^1^ Department of Pharmacy, COMSATS University Islamabad, Abbottabad, Pakistan; ^2^ Institute of Basic Medical Sciences, Khyber Medical University, Peshawar, Pakistan; ^3^ Cardiff School of Pharmacy and Pharmaceutical Sciences, Cardiff University, Cardiff, United Kingdom

**Keywords:** vincristine induced neuropathic pain, 5,7-Dimethoxycoumarin, monoamines in brain tissues, behavioral profiling, 5HT3 receptors, TNF-α, vitamin C

## Abstract

Vincristine is the drug of choice for Hodgkin’s lymphoma, acute lymphoblastic leukemia, and non-Hodgkin lymphoma. Despite its significant anticancer effects, it causes dose-dependent neuropathy, leading to compulsive dose reduction. The available drugs used for vincristine-induced neuropathic pain (VINP) have a range of safety, efficacy, and tolerability issues prompting a search for new therapies. 5,7-Dimethoxycoumarin (5,7-DMC) also known as citropten, is a natural coumarin found in the essential oils of citrus plants such as lime, lemons, and bergamots, and it possesses both antidepressant and anti-inflammatory effects. This study was designed to investigate the possible analgesic and antiallodynic effects of 5,7-DMC in a murine model of VINP. Vincristine was administered to groups of BALB/c male mice (0.1 mg/kg intraperitoneally) once daily for 14 days to induce VINP. Thermal hyperalgesia and mechanical allodynia were quantified using the tail immersion test and von Frey filament application method. The levels of monoamine neurotransmitters and vitamin C in frontal cortical, striatal and hippocampal tissues, as well as the TNF-α level in plasma, were quantified using high performance liquid chromatography and ELISA respectively. On day 15 of the protocol, acute treatment with 5,7-DMC clearly reversed VINP thermal hyperalgesia, mechanical static allodynia, mechanical dynamic allodynia, and cold allodynia. The activity of 5,7-DMC against hyperalgesia and allodynia was inhibited by pretreatment with ondansetron but not naloxone, implicating a 5-HT_3_ receptor involvement. VINP vitamin C levels were restored by 5,7-DMC in the frontal cortex, and changes in serotonin, dopamine, adenosine, inosine and hypoxanthine levels caused by vincristine were reversed either fully or partially. Additionally, the vincristine-induced rise in hippocampal serotonin, dopamine, inosine and striatal serotonin was appreciably reversed by 5,7-DMC. 5,7-DMC also reversed the vincristine-induced increase in the plasma level of TNF-α. In negating the changes in the levels of some neurotransmitters in the brain caused by vincristine, 5,7-DMC showed stronger effects than gabapentin. It was concluded that, there is a potential role of 5-HT3 receptors and monoamines in the amelioration of VINP induced by 5,7-DMC, and the use of this compound warrants further investigation.

## 1 Introduction

Neuropathic pain has been defined as pain caused by a lesion or disease of the somatosensory nervous system either centrally or peripherally (IASP, 2023). The symptoms may be linked with abnormal sensations termed dysesthesia (burning, coldness, paresthesia, numbness and itching), hyperalgesia (heightened pain sensitivity) or allodynia (perception of non-nociceptive stimuli as painful) and they may be spontaneous or evoked (Kaur et al., 2019).

The causes of neuropathic pain include diabetes, alcoholism, central nervous system damage, amputation, spinal nerve compression, nerve damage during surgery, nerve compression or infiltration by tumors, radiation therapy and chemotherapy with agents such as cisplatin, paclitaxel, and vincristine (VCR) (Suh, 2021). In regard to this, VCR is an effective anticancer drug used to treat Hodgkin’s and non-Hodgkin’s lymphoma, acute lymphoblastic leukemia, neuroblastoma, Wilms tumor, and rhabdomyosarcoma (Škubník et al., 2021; Barnett et al., 2022). It is the drug of choice for childhood blood cancers, but unfortunately, it can cause dose-dependent neuropathy, giving rise to dose limitation or even a complete discontinuation of therapy and treatment failure (Li et al., 2020). Peripheral neuropathy develops in 78% of patients and 44% patients receiving VCR have reported pain (Lavoie Smith et al., 2015). In another clinical study, 34.9% of patients experienced vincristine induced neuropathic pain VINP (Anghelescu et al., 2011). The clinical diagnostic features of VINP are typically variable (Li et al., 2020) and include gait dysfunction, constipation, weakness, urinary retention, tingling, paresthesia and loss of ankle stretch reflexes, some of the features being in a symmetrical, distal, “glove and stocking-like” distribution. The sensory symptoms of VINP are predominant with respect to severity and frequency compared with other chemotherapeutic drugs (Said and Tsimberidou, 2014; Madsen et al., 2019). However, the exact mechanism underlying VINP remains unclear. Nonetheless, VCR targets microtubules and impairs retrograde and anterograde neuronal transport and this has been suggested as an underlying cause of altered sensory neuronal function. Other proposed mechanisms include swelling of intracellular axonal mitochondria leading to the release of calcium ions and apoptosis; increased substance P levels in the spinal cord (Chiba et al., 2022), involvement of adenosine signaling in pain transmission (Zhou et al., 2022), increased release of substances including tumor necrosis factor-α (TNF-α), interleukin-1 [IL-1], interleukin-6 [IL-6] and nitric oxide [NO]) from glial cells, macrophages and Langerhans cells; as well as downregulation of IL-10 in spinal cord dorsal horns, increased nitric oxide synthase (NOS) in the dorsal horn, increased 5-hydroxytryptamine (5-HT2A) receptors on dorsal horn and dorsal root ganglion (DRG) neurons, increased reactive oxygen species (ROS) which affect serine protease activity and decreased endorphins in the spinal cord and DRG; and increased serine proteinase that inactivates endorphins (Thibault et al., 2008; Addington and Freimer, 2016). Moreover, it has been reported that L-ascorbic acid (vitamin C) reverses VCR induced astrocyte activation (Li G. Y. et al., 2021). It also potentiates the effects of vitamin E (Sisignano et al., 2014) in neuropathic pain and plasma vitamin C levels are lowered in patients with postherpetic neuralgia (Sekiguchi and Kawabata, 2013).

The impact of VCR on the central nervous system has been under active investigation as there are also other central derangements that may contribute to the development of VINP. In this regard, serotonin is considered to be a key neurotransmitter that contributes to pain control via descending pathways (Heijmans et al., 2021) and serotonin transporter knockout mice disclose less hyperalgesia but heightened neuropathic pain. In addition, serotonin at the cortical level and in mesolimbic system, has an inhibitory effect on pain, while in the spinal cord and the periphery, it is pro-nociceptive. What is more, serotonin receptors modulate both sensory and emotional components of pain, and 5-HT3 and 5-HT7 receptors have a bimodal role in controlling pain centrally (de Kort et al., 2022; Neugebauer, 2020).

Central dopaminergic activation in the periaqueductal grey/dorsal raphe system induces anxiety-free analgesia, and oxytocin release onto target cells in the ventrolateral periaqueductal grey, inhibits spinal cord sensory neurons, producing antinociception thought to be critical in neuropathic pain models (Taylor et al., 2019; Iwasaki et al., 2023). In addition to this, both dopamine itself and D2 receptors in the striatum and spinal cord are implicated in generating highly effective analgesia (Wang et al., 2021). Furthermore, overexpression of monoamine oxidase B in the spinal cord and dorsal root ganglion has been reported during chemotherapy induced neuropathy, whilst inhibition of this enzyme ameliorates the overall pathology (Ouyang et al., 2023). Additionally glial α7 nAChR activation by agonists, contributes towards amelioration of pain and inflammation both centrally and peripherally (Hone and McIntosh, 2023).

In a chemotherapy induced neuropathy model, descending noradrenergic inhibition in the spinal cord is elevated, prompting the suggestion that chemotherapy augmented noradrenergic inhibition may stem from an adaptive mechanism to increased peripheral nociceptive input (Costa-Pereira, Ribeiro, Martins and Tavares, 2020; Sałat, 2020; Song et al., 2020). In addition to this, during chemotherapy induced peripheral neuropathy, adenosine 1A receptor expression is also suppressed in the spinal cord (Liu et al., 2023).

VCR tends to produce greater neurotoxicity compared to other Vinca alkaloids (Grisold et al., 2012), and this may be ascribed to microtubule destabilization during mitosis (metaphase) and interference with axonal transport (Stubblefield et al., 2009; Nie et al., 2017; Triarico et al., 2021). Currently, there is no standard treatment available for VINP, rather than merely managing it (Kanzawa-Lee et al., 2019). Presently, the anti-epileptics (pregabalin and gabapentin), tricyclic antidepressants (amitriptyline), a serotonin-noradrenaline reuptake inhibitor (duloxetine), selective serotonin reuptake inhibitors (fluoxetine, citalopram and paroxetine), an opioid agonist (tramadol), an NMDA antagonist (ketamine), pyridoxine and pyridostigmine, are used in different combinations for the management of complex neuropathic pain (Stubblefield et al., 2009; Addington and Freimer, 2016; Aydin Köker et al., 2021; Triarico et al., 2021). Such therapies modify serotonergic, adrenergic, excitatory amino acid or opioidergic pathways to produce anti-allodynic effects with unsatisfactory clinical outcomes associated with serious adverse effects, including dependence (Wiffen et al., 2017; Cho et al., 2021). Considering the complex nature of neuropathic pain, and the serious efficacy and tolerability issues of currently available therapies, there is an ongoing search for potential molecules, both natural and synthetic (Chung and Kim, 2022), for the treatment of VINP.

In relation to this quest, naturally occurring coumarins consist of fused benzene and pyrone rings (Common and IUPAC names: benzopyran-2-one and chromen-2-one). These compounds exhibit low toxicity, good bioavailability, high potency, and a broad-spectrum activity against various diseases. Natural coumarins have antioxidant, neuroprotective, anti-inflammatory, antidiabetic, antidepressant, and anticonvulsant properties, whereas methoxycoumarins possess antioxidant and anti-Alzheimer’s disease properties (Marchant et al., 1985; Zhu and Jiang, 2018; Mishra et al., 2020; Chaurasiya et al., 2022; Keri et al., 2022). 5,7-Dimethoxycoumarin (5,7-DMC) also known as citropten, is an example of naturally occurring methoxy coumarins found in Carica papaya, Citrus aurantifloia, Pelea anisata H. Mann and Bergamot (Citrus bergamia, Risso), and it has an established antidepressant-like activity (Alesiani et al., 2008; Mishra et al., 2020). However, the pharmacological potential of 5,7-DMC in VINP, its interaction with opioidergic or serotonergic pathways, and associated changes in brain neurotransmitters in addition to vitamin C levels, has not been explored. The purpose of this study therefore, was to examine the pharmacological potential of 5,7-DMC in a model of VCR-induced neuropathic pain. The investigation was focused on any possible mechanism of action through its interaction with opioidergic or serotonergic pathways and the associated changes in the concentrations of vitamin C and neurotransmitters in brain areas, as well as the level of TNF-α in plasma.

## 2 Materials and methods

### 2.1 Ethical Approval

Experimental procedures were approved by the Research Ethical Committee, COMSATS University Islamabad, Abbottabad Campus, Abbottabad, Khyber Pakhtunkhwa, Pakistan (Ethical Approval number: PHM. Eth/CS-M01-017–1037) and complied with the United Kingdom Animals (Scientific Procedures) Act 1986.

### 2.2 Sources of the TNF-α ELISA kit, 5,7-DMC and its vehicle

The TNF-α ELISA kit was purchased from the Cayman Chemical Company, Ann Arbor, MI, United States 5,7-DMC was purchased from Biosynth, United Kingdom Unit 8 & 9 Old Station Business Pk, Compton RG20 6NE, United Kingdom. A uniform and homogeneous suspension of 5,7-DMC was prepared in 1% carboxymethylcellulose.

### 2.3 Experimental animals

Male BALB/c mice weighing 21–25 g were maintained at 23–25°C on a 12–12 h light-dark cycle, and they were provided with *ad libitum* access to food and water.

The animals were divided into the following treatment groups (*n* = 6/group):

a) Vehicle daily (10 mL/kg, intraperitoneally [i.p.])
b) VCR daily (0.1 mg/kg, i. p.)
c) VCR daily (0.1 mg/kg, i. p.) + gabapentin (75 mg/kg, i. p.)
d) VCR daily (0.1 mg/kg, i. p.) + 5,7-DMC (30 mg/kg, i. p.)
e) VCR daily (0.1 mg/kg, i. p.) + 5,7-DMC (40 mg/kg, i. p.)
f) VCR daily (0.1 mg/kg, i. p.) + 5,7-DMC (50 mg/kg, i. p.)

**FIGURE 1:**
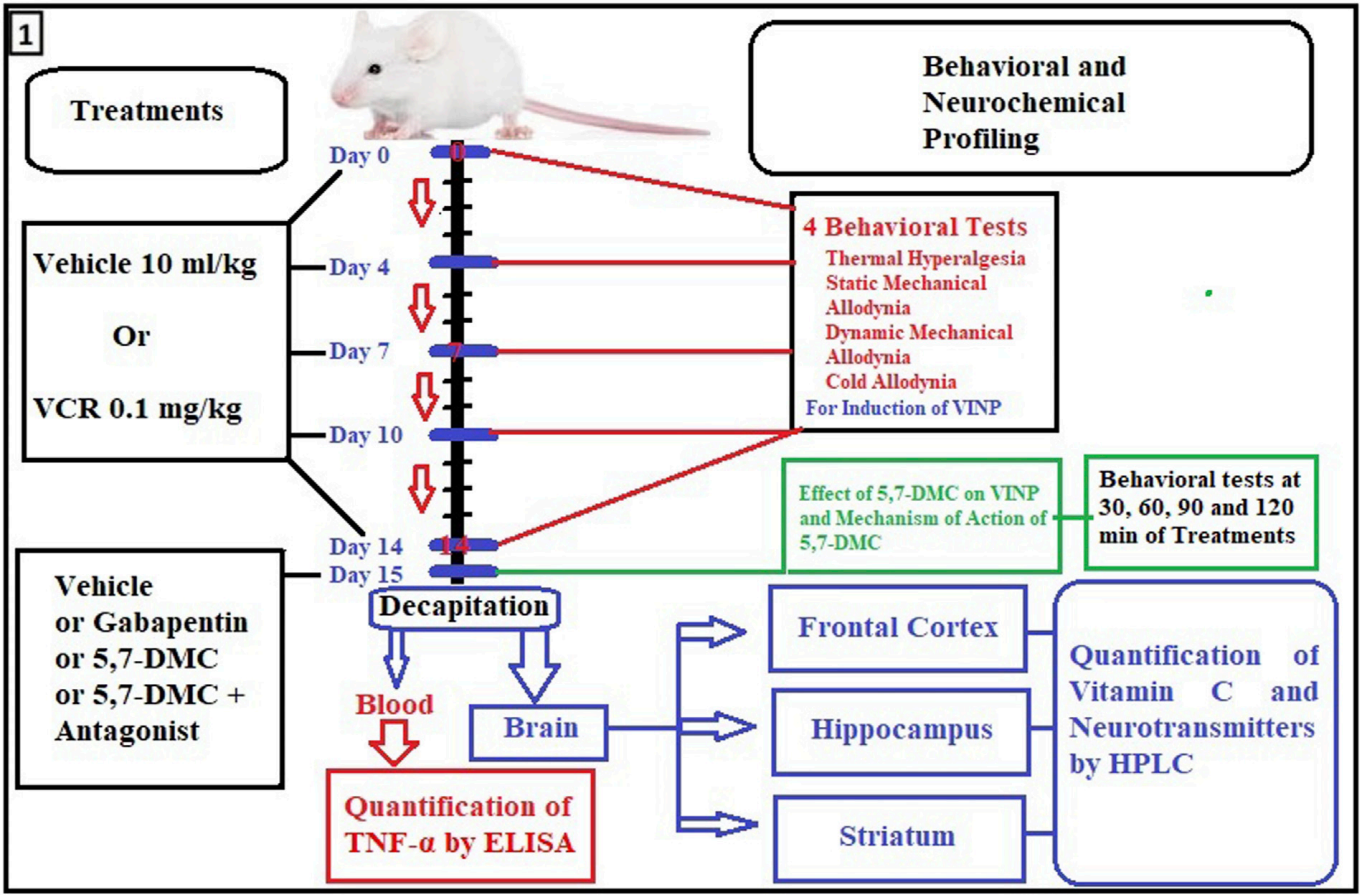
Flow diagram showing timing details of dosing, days and experimental procedures including blood and brain tissue collection. For assessment of VINP, behavioral tests were performed on day 0, 4, 7, 10 and 14. Moreover, behavioral tests, quantification of TNF-α, mechanism of action of 5,7-DMC and neurochemical analyses were performed on the 15th protocol day by the acute administration of 5,7-DMC or gabapentin.

The 14-day schedule of dosing and experimental protocol, including blood and brain tissue collection, is shown in Figure 1. On day 15, a single acute dose of either 30, 40 or 50 mg/kg of 5,7-DMC, or 75 mg/kg of gabapentin was administered to the independent groups and behavioral readings were performed after 30, 60, 90 and 120 min of treatment, there being no overt signs of motor discoordination.

### 2.4 Induction of VINP

VCR was injected i. p. once daily at a dose of 0.1 mg/kg subacutely for 14 consecutive days. Behavioral tests (tail immersion, cold allodynia, von Frey filament static pressure and dynamic allodynia tests) were performed on days 0, 4, 7, 10, and 14 of the VCR treatment protocol (Zhu et al., 2021).

### 2.5 Behavioral tests

Separate parallel animal groups were subjected to only one of the behavioral tests at a time, rather than multiple sequential tests on the same animals. Consequently, individual animals were exposed to only a single nociceptive stimulus type in order to avoid any influence of one test upon another.

#### 2.5.1 Thermal hyperalgesia

Thermal hyperalgesia was evaluated using the tail immersion test. Mice were positioned in light restraining cages in such a way that the tail protruded. Animals were habituated to their surroundings 30 min before experimentation. The lower 3 cm portion of the tail was immersed in water maintained at 54°C ± 0.5°C. A tail withdrawal latency (TWL) was measured in seconds (s). Readings in triplicate were taken, and a 15 s cut-off period was imposed to prevent any tissue injury (Sewell and Spencer, 1976; Nawaz et al., 2018; Khan et al., 2019).

#### 2.5.2 Static mechanical allodynia

Mechanical allodynia was gauged by using von Frey filaments. Mice were placed in cages having dimensions of 30 cm × 24 cm (*H*×*D*) with a fine metal mesh (1.3 cm × 1.3 cm apertures) to allow direct access to mice paws from below (Bardin et al., 2009). Prior to each experiment, mice were habituated with the experimental conditions. Von Frey filaments of different ranges were applied to the left hind paw for 2 s. If mice responded to a selected filament, then a lower force was used, but if mice did not respond to the selected filament, then higher force was applied. This method was repeated four times after the first response towards a filament. A cut-off time of 6 s was imposed to measure the pain threshold (Chaplan et al., 1994; Shahid et al., 2017).

#### 2.5.3 Dynamic mechanical allodynia

Following a 30 min habituation period, dynamic allodynia was evaluated using a cotton bud which was lightly brushed on the plantar surface area of the hind paws. Abrupt paw withdrawal, flinching or licking, was evaluated as a paw reaction. Mice that responded to brushing within 8 s were included in the test. Readings in triplicate were taken and a 15 s cut-off period was imposed (Shahid et al., 2017; Khan et al., 2019).

#### 2.5.4 Acetone drop-induced cold allodynia

Mice were placed on wire netting bottomed cages for a habituation period of 10 min. A 50 µL drop of acetone was applied to each hind paw using a syringe connected to a blunt needle. The paw withdrawal response to the cooling effect of each acetone drop was noted within a minimum period of 0.5 s and a maximum of 15 s (Shahid et al., 2017; Khan et al., 2019). The response was classed into four categories (0 = no response; 1 = brisk withdrawal or flick of the paw; 2 = repeated flicking of the paw; 3 = repeated flicking and licking of the paw). Acetone was applied in triplicate to each hind paw and a mean value was calculated (Bardin et al., 2009; Deuis et al., 2017).

### 2.6 Mechanism of 5,7-DMC analgesic or antiallodynic effects

The possible mechanism of the analgesic and antiallodynic effects of 5,7-DMC in VINP was explored by examining the role of opioidergic or serotonergic pathways using specific receptor-specific antagonists (naloxone: opioid antagonist or ondansetron: 5HT3 antagonist) and the animals were pretreated (10 min) with a 1.0 mg/kg dose of each antagonist (Ali et al., 2022; Tokhi et al., 2023) and then 5,7-DMC was administered.

### 2.7 Quantification of TNF-α in plasma

Blood was collected from mice after decapitation by direct cardiac puncture. It was centrifuged and the plasma was kept at the specified temperature stipulated by the manufacturer of the ELISA kit, and the TNF-α concentration was quantified according to the manufacturer’s instructions (Zhou et al., 2018).

### 2.8 Neurochemical analysis

#### 2.8.1 Sample preparation

Following behavioral assessment, post-mortem (by decapitation) whole brains were removed and frontal cortical, striatal, and hippocampal tissues were dissected on ice chilled plates, precisely weighed, and kept at −80°C. Homogenization of the tissues was carried out in chilled perchloric acid (0.2%) following cold centrifugation (4°C; 12,000 rpm) and the supernatant was then separated. Once the samples were prepared, they were passed through a 0.45 mm filter and then analyzed using HPLC (Ur Rehman et al., 2020; Arif et al., 2022).

#### 2.8.2 Chromatographic conditions

A Waters Alliance 2,690 separation module with UV detector was used for the chromatographic analysis (United States) coupled to a C18 stainless steel column with a 5 µm particle size (250 × 4.6 mm). For vitamin C, dopamine and noradrenaline analysis, methanol, HPLC grade water and 20 mM monobasic sodium phosphate (5:95, v/v) were used with isocratic elution at 280 nm at a column temperature of 35°C and an elution rate of 0.5 mL/min. For the analysis of adenosine, inosine and hypoxanthine, acetonitrile, HPLC grade water and 0.01 M monobasic sodium phosphate (5:95 v/v) was used with isocratic elution at 260 nm, with the column at room temperature and a flow rate of 1.0 mL/min (Ur Rehman et al., 2020; Arif et al., 2022).

#### 2.8.3 Standard preparation

Standard stock solutions were made by dissolving 1.0 mg of each of the following compounds in 10 mL of HPLC-grade water: vitamin C, dopamine, 5-HT, noradrenaline, adenosine, inosine, and hypoxanthine. The standard stock solution was then diluted to provide various concentrations ranging from 100 to 500 ng/mL from which, calibration curves were constructed. The samples were loaded into the HPLC system, and a 20 µL sample volume was extracted for injection (EmpowerTM). Then, using linear regression analysis, a calibration curve was plotted against the peak area of sample (y) and the concentration (x) (Ur Rehman et al., 2020; Arif et al., 2022; Tokhi et al., 2023).

### 2.9 Statistical analysis

Data was expressed as mean ± SEM (*n* = 6/group) and analyzed statistically with the assistance of Graph Pad Prism 8. One-way ANOVA with *post hoc* Tukey’s test (multiple comparisons) was used to determine the level of significance. The non-parametric Mann-Whitney test was applied for comparing scores of two groups and Kruskal–Wallis test was performed for multiple comparisons. Two-way ANOVA was used to determine the level of significance for the data involving time (30, 60, 90 and 120 min).

## 3 Results

### 3.1 5,7-DMC reduces VCR-induced thermal hyperalgesia and cold allodynia in a dose-dependent manner

**FIGURE 2:**
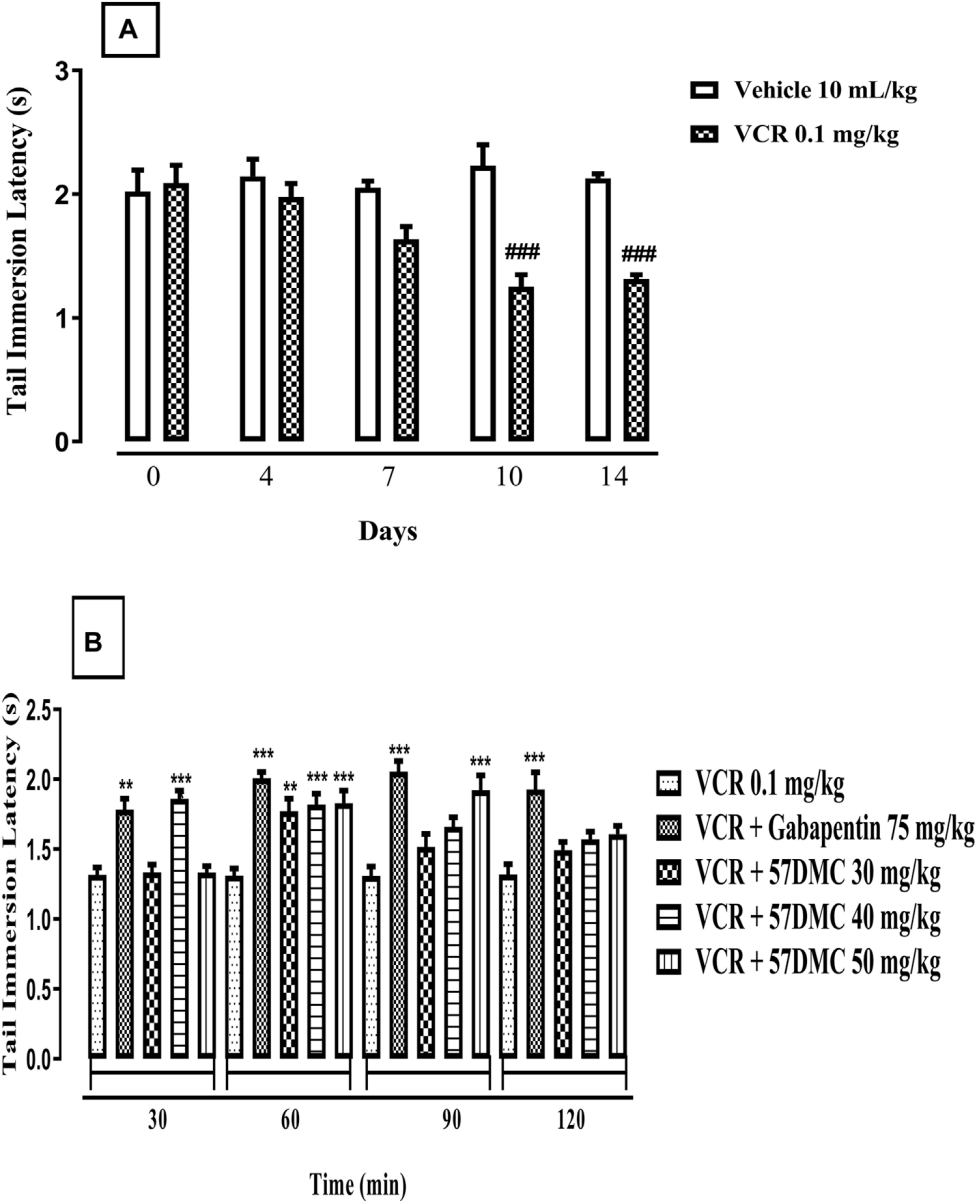
**(A)** Development of VCR-induced thermal hyperalgesia in the mouse tail immersion test. The significance of reduced tail immersion latencies (s) (mean ± SEM), are compared with the vehicle treated control ###*p* < 0.001; confidence interval: 95%; n = 6; one way ANOVA with *post hoc* Tukey’s test. **(B)** Effect of acute treatment with 5,7-DMC or gabapentin on vincristine (VCR) induced thermal hyperalgesia in the mouse tail immersion test. The significance of differences in tail immersion latencies (s) (mean ± SEM), are presented *versus* the vincristine treated group: **p < 0.01; ***p < 0.001; confidence interval: 95%; n = 6; test applied: Two-way ANOVA with *post hoc* Tukey’s test.

Treatment with VCR (0.1 mg/kg) daily for 14 days resulted in the development of thermal hyperalgesia indicated by a notable decrease in mouse tail immersion latency on days 10 (*p* < 0.0001) and 14 (*p* = 0.0004) in comparison with the vehicle treated animal group (F (9, 50) = 9.165, *p* < 0.0001) (Figure 2A). One-way ANOVA (with *post hoc* Tukey’s test) was applied. 5,7-DMC reversed VCR-induced thermal hyperalgesia at 30 mg/kg, 60 min after administration while at 40 mg/kg the reversal was significant at 30 and 60 min. The 50 mg/kg dose of 5,7-DMC reversed the VCR-induced hyperalgesia 60 and 90 min after administration. Gabapentin (positive control) at a higher dose (75 mg/kg), produced a notable improvement in response latency throughout the testing phase as compared to the VCR treated hyperalgesic group. Two-way ANOVA was applied [row factor: F (5, 95) = 2.424, *p* = 0.041; column factor: F (19, 95) = 13.06, *p* < 0.0001] (Figure 2B).

**FIGURE 3:**
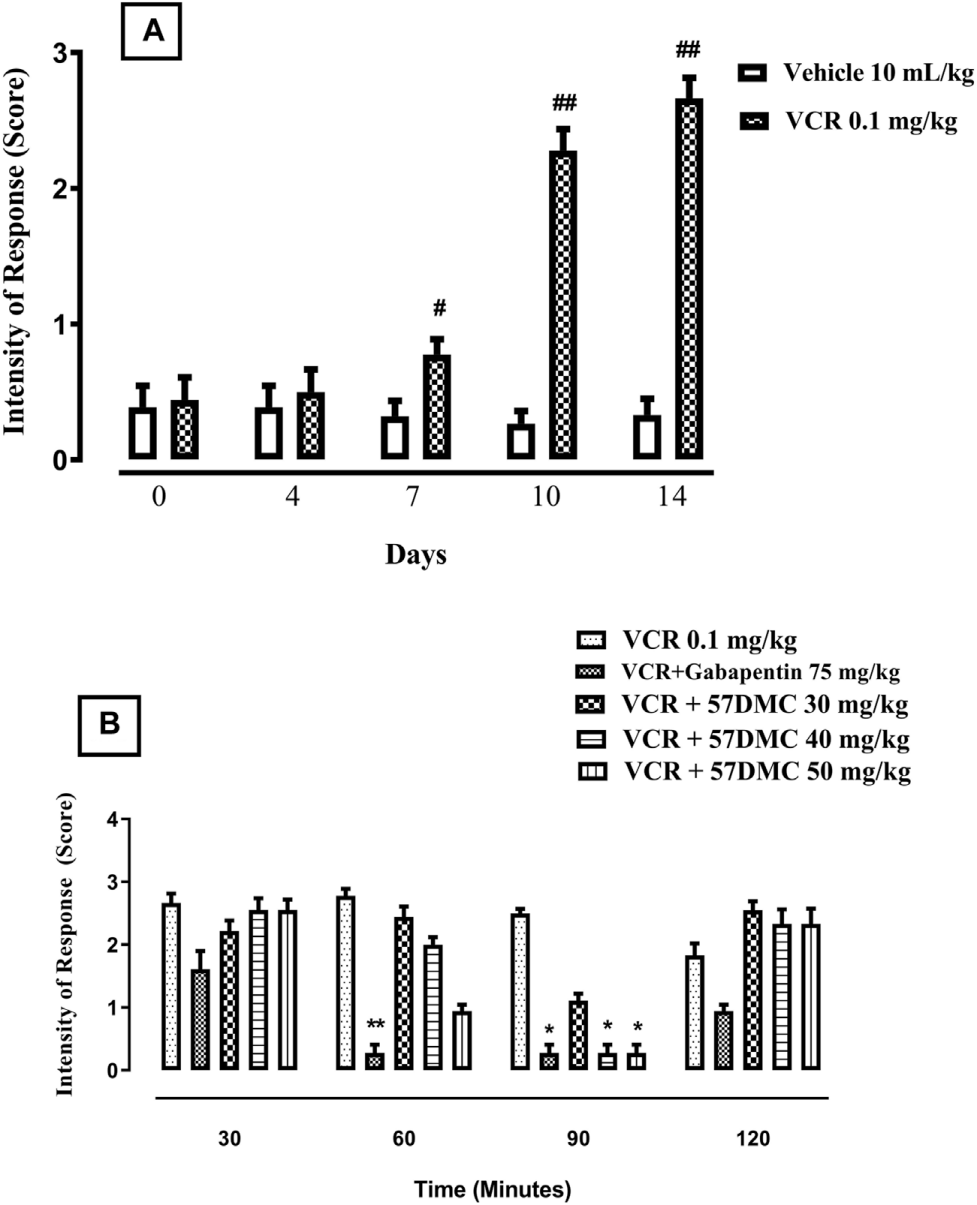
**(A)** Development of VCR cold allodynia signified by an increased response score to application of acetone on the mouse paw plantar surface. The significance of increased response scores (mean ± SEM), are compared with vehicle treated control: #*p* < 0.05 and ###*p* < 0.001; confidence interval: 95%; n = 6 (Mann-Whitney test). **(B)** Effect of acute treatment with 5,7-DMC or gabapentin on VCR induced cold allodynia in response to mouse paw plantar surface application of acetone. The significance of differences in response scores (mean ± SEM) are presented *versus* the VCR treated group: *p < 0.05, **p < 0.01 and ***p < 0.001; confidence interval: 95%; *n* = 6 (Kruskal–Wallis test).

The 14-day VCR treatment protocol generated cold allodynia, shown by a steady increase in the intensity of response score in response to acetone application from days 7(*p* = 0.0281), 10 (*p* = 0.0022) and 14 (*p* = 0.0022) of the protocol onwards (Mann-Whitney test) (Figure 3A). 5,7-DMC significantly reversed VCR-induced cold allodynia in response to mouse paw acetone application. The 5,7-DMC reversal was substantiated by a reduced intensity response score. After 60 min, the reversal was not significant with any dose (*p* > 0.99), and after 90 min both the 40 and 50 mg/kg doses maintained the VCR cold allodynia reversal (*p* = 0.0389). Gabapentin also reversed the VCR-induced cold allodynia at 60 and 90 min of the test period (*p* = 0.017 and *p* = 0.0389) (Kruskal–Wallis test) (Figure 3B).

### 3.2 5,7-DMC reduces VCR-induced static mechanical allodynia and dynamic mechanical allodynia in a dose-dependent manner

**FIGURE 4:**
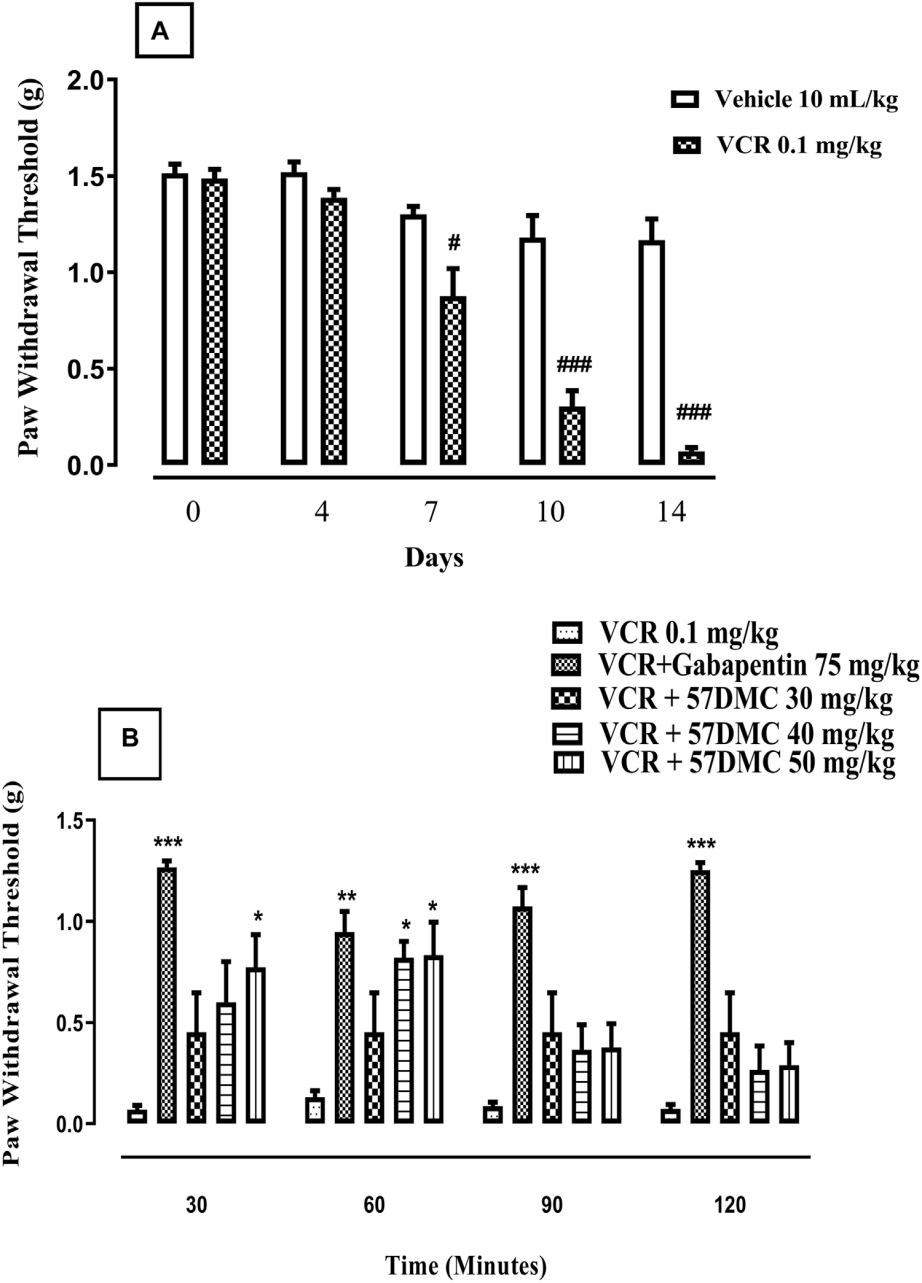
**(A)** Development of VCR-induced static mechanical allodynia in the von Frey filament mouse paw withdrawal threshold test. The significance of reduced paw withdrawal thresholds (g) (mean ± SEM), are compared with vehicle treated control: #*p* < 0.05 and ###*p* < 0.001; confidence interval: 95%; *n* = 6; one way ANOVA with *post hoc* Tukey’s test. **(B)**- Effect of acute treatment with 5,7-DMC or gabapentin on vincristine (VCR) induced static mechanical allodynia in the mouse paw withdrawal pressure threshold test. The significance of differences in paw withdrawal thresholds (g) (mean ± SEM), are presented *versus* the VCR treated group: *p < 0.05, **p < 0.01 and ***p < 0.001; confidence interval: 95%; *n* = 6; two-way ANOVA with *post hoc* Tukey’s test.

VCR daily treatment in the 14-day protocol, led to the development of static allodynia as evidenced by decreased paw withdrawal thresholds from treatment day 7 (*p* < 0.05), 10 (*p* < 0.001) and 14 (*p* < 0.001) in comparison with controls (F (9, 50) = 41.1, *p* < 0.0001) (Figure 4A). One-way ANOVA (with *post hoc* Tukey’s test) was applied. There was a substantial improvement (i.e., elevation of paw withdrawal threshold), in VCR-induced mechanical allodynia produced by 5,7-DMC at the two higher doses at 60 min and at the 50 mg/kg dose, 30 min after its administration. Similarly, gabapentin reversed the VCR provoked suppression of paw withdrawal threshold up to 2 h after dosing. Two-way ANOVA was applied [row factor: F (5, 95) = 8.097, *p* < 0.0001; column factor: F (19, 95) = 11.82, *p* < 0.0001] (Figure 4B).

**FIGURE 5:**
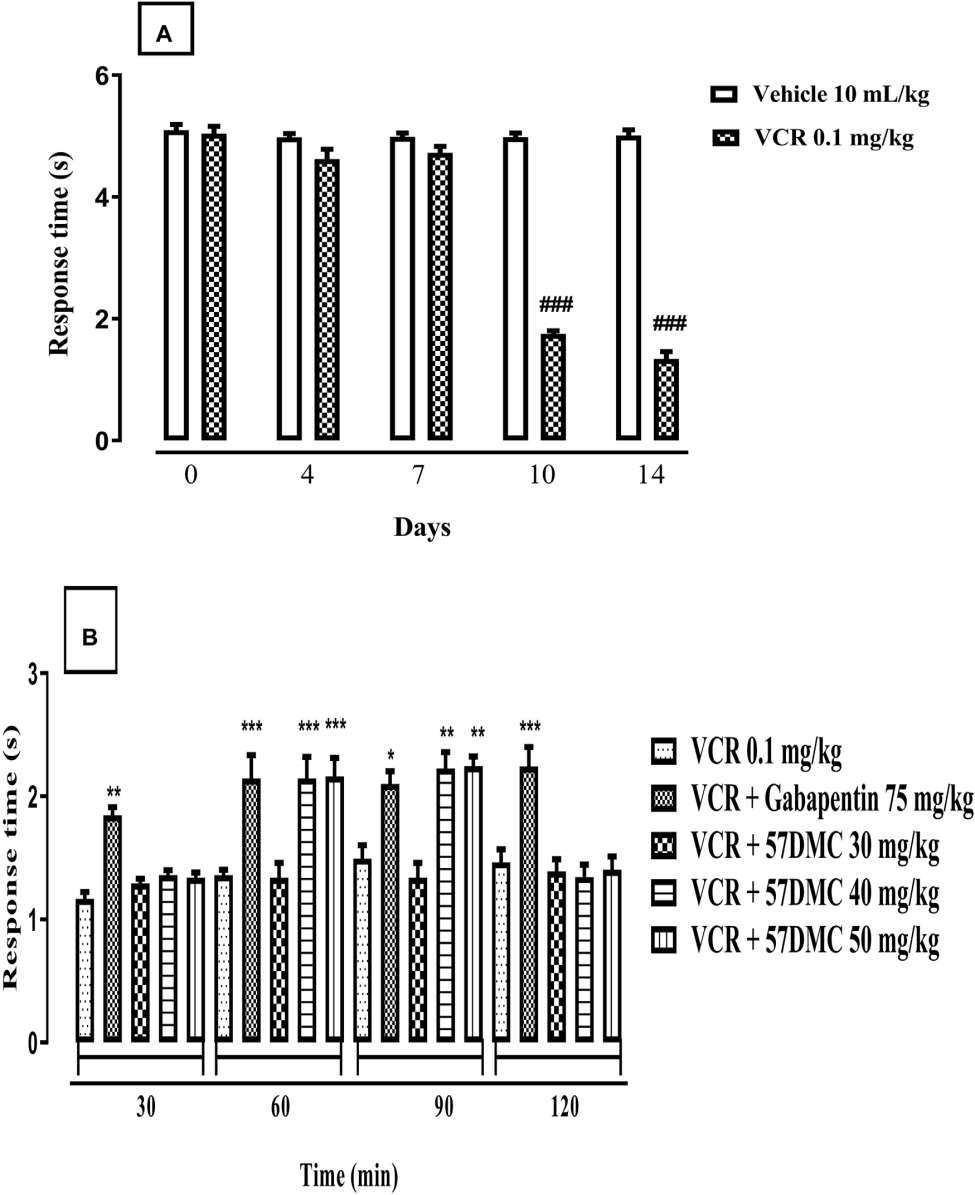
**(A)** Development of VCR induced dynamic allodynia revealed by a decreased response time (s) in mice following light brushing of the plantar surface of their hind paws. The significance of reduced response latencies (s) (mean ± SEM), are compared with the vehicle treated control: ###*p* < 0.001; confidence interval: 95%; *n* = 6; One way ANOVA with *post hoc* Tukey’s test. **(B)** Effect of acute treatment with 5,7-DMC or gabapentin on vincristine (VCR) induced dynamic mechanical allodynia in response to light brushing of mouse paw plantar surface. The significance of differences in response latencies (s) (mean ± SEM), are presented *versus* the VCR treated group: *p < 0.05, **p < 0.01 and ***p < 0.001; confidence interval: 95%; *n* = 6; Two-way ANOVA with *post hoc* Tukey’s test.

Fourteen-day treatment with VCR gave rise to the development of dynamic allodynia revealed by a decrease in response time following light brushing of mouse paw. The onset of effect occurred on days 10 (*p* < 0.001) and 14 (*p* < 0.001) in comparison with controls (F (9, 50) = 208.7, *p* < 0.0001) (Figure 5A). One-way ANOVA (with *post hoc* Tukey’s test) was applied. 5,7-DMC ameliorated VCR-induced dynamic mechanical allodynia 60–90 min after administration of the 40–50 mg/kg doses, which caused increased response times to light plantar paw brushing. Likewise, gabapentin evoked an increase in response times throughout the whole 120 min testing period after injection of this positive control *versus* the VCR treated group. Two-way ANOVA was applied [row factor: F (5, 95) = 12.29, *p* < 0.0001; column factor: F (19, 95) = 20.90, *p* < 0.0001] (Figure 5B).

### 3.3 Involvement of a serotonergic but not opioidergic mechanism in the anti-hyperalgesic and anti-allodynic activity of 5,7-DMC

**FIGURE 6:**
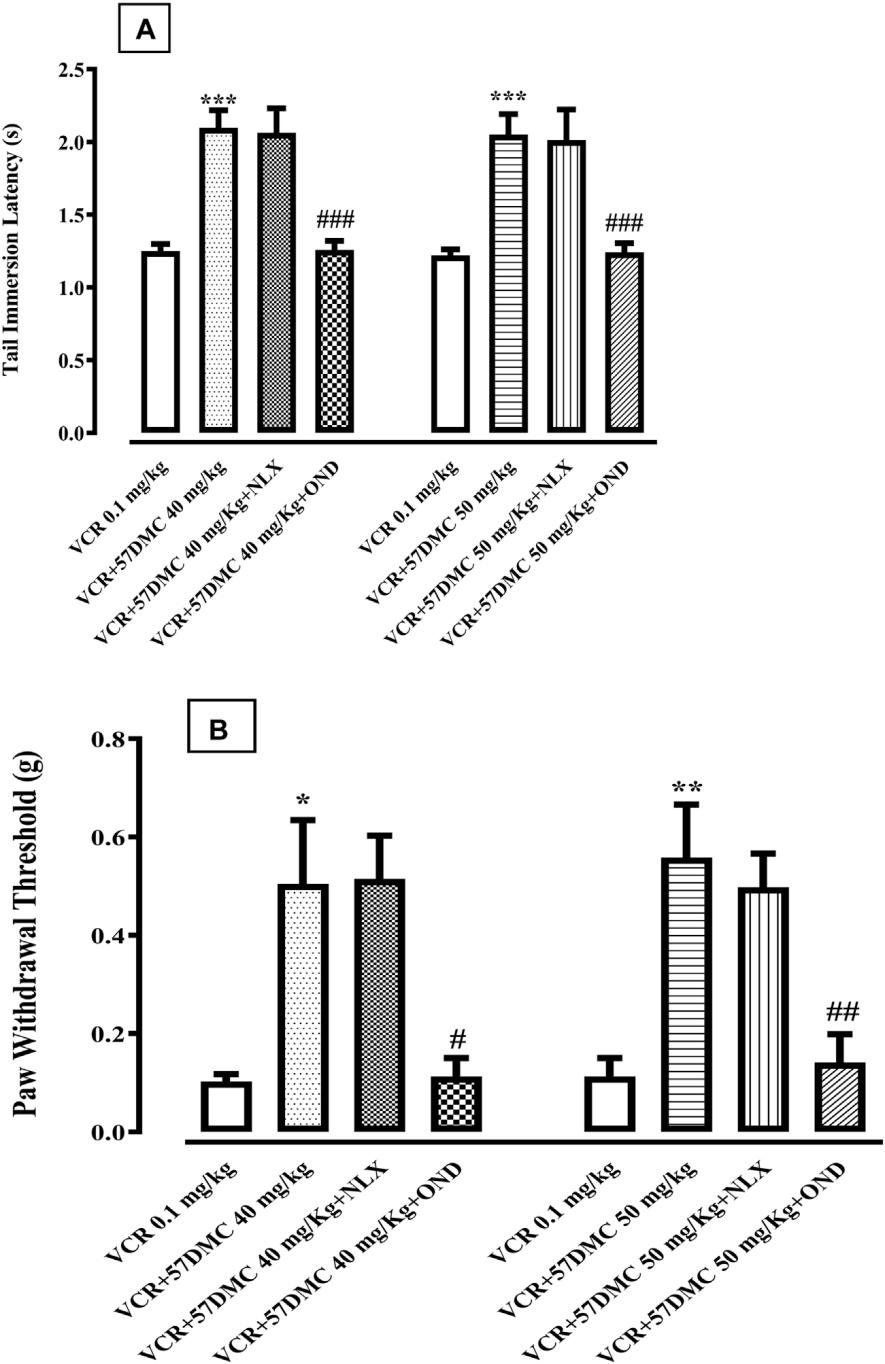
**(A)** Effect of naloxone (Nlx) or ondansetron (Ond) 10 min pretreatment on 5,7-Dimethoxycoumarin (5,7-DMC) activity against vincristine (VCR) induced hyperalgesia in the mouse tail immersion test. The significance of differences in latencies (s) (mean ± SEM) are presented *versus* the VCR treated group: ***p < 0.001; significance of differences (s) (mean ± SEM) are presented *versus* the 5,7-DMC treated VCR group: ###*p* < 0.001; confidence interval: 95%; *n* = 6; one way ANOVA with *post hoc* Tukey’s test. **(B)** Effect of ondansetron (Ond) or naloxone (Nlx) 10 min pretreatment on 5,7-Dimethoxycoumarin (5,7-DMC) activity against vincristine (VCR) induced allodynia in the mouse paw pressure test. The significance of threshold differences (g) (mean ± SEM) are presented *versus* the VCR treated group: *p < 0.05, **p < 0.01, ***p < 0.001; significance of threshold differences (g) (mean ± SEM) are presented *versus* the 5,7-DMC treated VCR group: ###*p* < 0.001, ##*p* < 0.01; confidence interval: 95%; *n* = 6; one way ANOVA with *post hoc* Tukey’s test.

5,7-DMC (40 and 50 mg/kg) reversed VCR-induced thermal hyperalgesia, by increasing the tail-immersion latency, and this reversal was not inhibited by 10 min pretreatment with the naloxone (1.0 mg/kg). However, the anti-hyperalgesic effect of both doses of 5,7-DMC, was significantly inhibited by 10 min pretreatment with ondansetron (1.0 mg/kg), reducing the tail immersion latency to a comparable level with the VCR protocol group [F (7, 40) = 12.73] (Figure 6A). One-way ANOVA (with *post hoc* Tukey’s test) was applied.

5,7-DMC at doses of 40 and 50 mg/kg, reversed VCR-induced mechanical static allodynia by increasing the threshold pressure for paw withdrawal. This anti-allodynic effect of 5,7-DMC was antagonized by ondansetron (1.0 mg/kg), whereby the resultant allodynic response was comparable to that of the VCR protocol group. Naloxone in contrast, did not modify the anti-allodynic activity of 5,7-DMC [F (7, 40) = 7.863] (Figure 6B). One-way ANOVA (with *post hoc* Tukey’s test) was applied.

### 3.4 5,7-DMC reverses the vincristine-induced rise in plasma level of TNF-α

**FIGURE 7:**
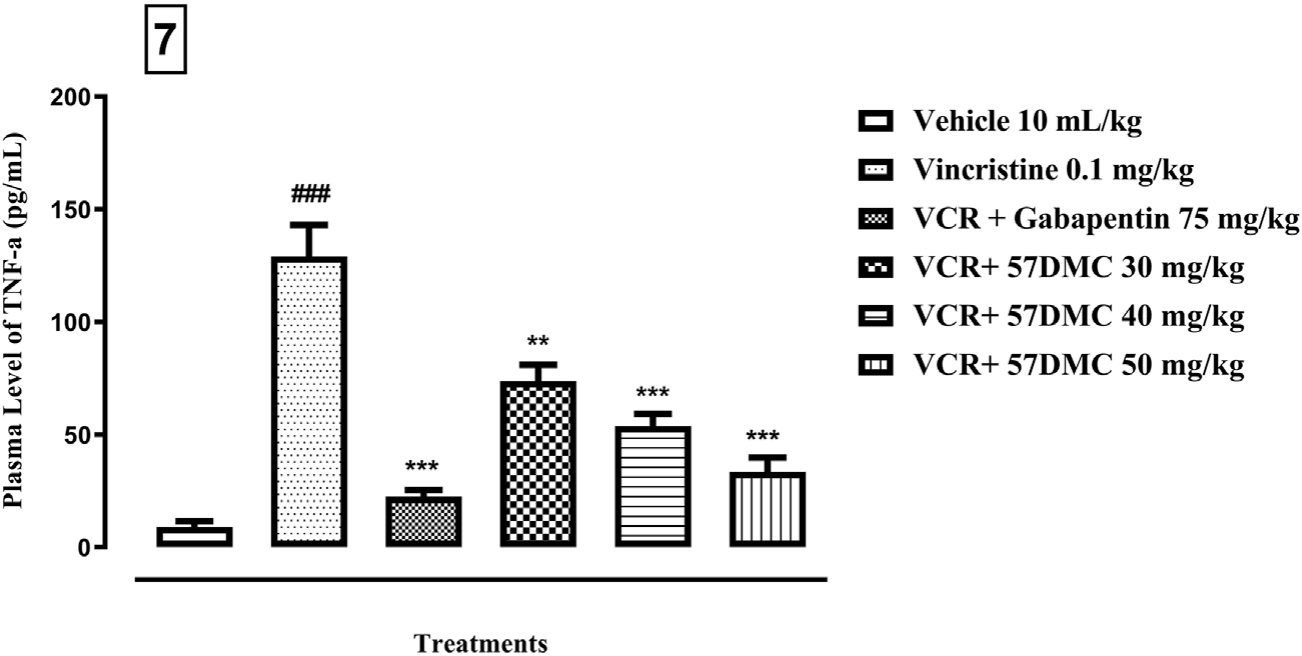
**-** Effect of acute treatment with 5,7-DMC or gabapentin on the level of TNF-α in plasma. The mean ± SEM values are expressed in pg/mL. The significance of differences are presented *versus* the VCR treated group: **p < 0.01, ***p < 0.001; significance of difference are presented *versus* the vehicle treated group: ###*p* < 0.001; confidence interval: 95%; *n* = 6; one way ANOVA with *post hoc* Tukey’s test.

As shown in Figure 7, the plasma level of TNF-α was increased (9.1 ± 2.5 up to 129.1 ± 14.0 pg/mL; *p* < 0.001) after the development of neuropathic pain, i.e., following administration of VCR (0.1 mg/kg i. p.), and this was reversed by gabapentin (129.1 ± 14.01 down to 22.4 ± 2.9; *p* < 0.001) and 5,7-DMC at doses of 30, 40 and 50 mg/kg (129.1 ± 14.01 down to 73.77 ± 7.22 *p* < 0.01, 53.77 ± 5.49 *p* < 0.001 and 33.4 ± 6.4 pg/mL *p* < 0.001 respectively) [F (5, 12) = 34.03] *when one-way ANOVA* (with Tukey’s test in *post hoc* analysis) was applied.

### 3.5 5,7-DMC reverses vincristine-induced changes in frontal cortical levels of monoamine neurotransmitters and vitamin C without affecting adenosine

Vitamin C levels were significantly brought down after the development of VINP, and this was significantly improved by 5,7-DMC (50 mg/kg) while gabapentin was inactive in this respect [*F* (*5*, *21*) = *0.7460*]. An upsurge in serotonin levels was observed after the development of VINP, and 5,7-DMC (40 and 50 mg/kg) notably repressed it back, while gabapentin caused a significant decrease *versus* the vehicle group [*F* (*5*, *16*) = *1.125*]. The noradrenaline level was also repressed by VCR, while 5,7-DMC treatment (30 and 40 mg/kg) increased the suppressed noradrenaline levels, but gabapentin had no significant effect [*F* (*5*, *15*) = *1.545*]. There were upsurges in dopamine levels after VCR treatment that were repressed by 5,7-DMC (30 and 50 mg/kg), but gabapentin actually enhanced the dopamine level [*F* (*5*, *16*) = 0*.7621*] *when one-way ANOVA* with *post hoc* Tukey’s test was applied. In addition, VCR induced neuropathy reduced adenosine levels in the frontal cortex, and the suppressed adenosine levels were elevated by the two higher doses of 5,7-DMC, while they were not changed by gabapentin [*F* (*5*, 2*1*) = 0*.7794*]. However, the inosine levels were not changed significantly, and they were improved significantly only by the highest dose of 5,7-DMC (50 mg/kg) [*F* (*5*, *19*) = 1*.905*]. An elevation in hypoxanthine levels was also seen in VINP, and it was subsequently lowered by the two highest doses of 5,7-DMC [*F* (*5*, *17*) = 0*.2186*] (Table 1), and they were further elevated by gabapentin in comparison with the vehicle group (*one-way ANOVA* with *post hoc* Tukey’s test).

**TABLE 1:** Effect of acute treatment with 5,7-DMC or gabapentin (GBP) on vincristine (VCR) induced changes in frontal cortical levels of vitamin C, neurotransmitters and two metabolites of adenosine: One way ANOVA with Tukey’s test was applied. The values (nanogram/mg of wet tissue) are expressed as mean ± SEM, confidence interval = 95% and the significance of differences from the vehicle and VCR group are #, ## and ### and *, ** and *** respectively for *p* < 0.05, *p* < 0.01 and *p* < 0.001.

	Vehicle	VCR	VCR + GBP	VCR +5,7-DMC	VCR +5,7-DMC	VCR +5,7-DMC
	10 mL/kg	0.1 mg/kg	75 mg/kg	30 mg/kg	40 mg/kg	50 mg/kg
**Vitamin C**	12.98 ± 1.07	7.12 ± 1.06###	6.62 ± 0.38	9.73 ± 0.67	8.70 ± 2.78	13.78 ± 1.41***
**Serotonin**	9.52 ± 1.82	30.4 ± 5.0###	1.12 ± 0.22***	21.13 ± 2.6	12.03 ± 1.41*	8.87 ± 0.86**
**Norepinephrine**	13.15 ± 1.36	5.75 ± 0.76##	6.9 ± 0.33	24.47 ± 6.35***	57.38 ± 6.32***	1.8 ± 0.17***
**Dopamine**	9.08 ± 2.92	69.27 ± 6.81###	97.18 ± 10.95 *	57.6 ± 5.73*	78.7 ± 9.72	25.02 ± 4.46 ***
**Adenosine**	14.5 ± 1.65	5.48 ± 0.36###	8.58 ± 1.06	3.68 ± 0.57	6.16 ± 0.94	16.72 ± 2.83***
**Inosine**	34.58 ± 1.75	33.55 ± 9.3	13.28 ± 0.94	17.35 ± 1.17	43.08 ± 8.61	119.8 ± 5.38***
**Hypoxanthine**	23.66 ± 1.14	77.2 ± 10.5###	96.15 ± 4.16*	93.63 ± 17.35	29.56 ± 10.67**	13.88 ± 2.49***

### 3.6 5,7-DMC reverses vincristine-induced changes in hippocampal levels of neurotransmitters without affecting vitamin C and noradrenaline

The increased serotonin level in the hippocampus after the development of VINP, was notably lowered by 5,7-DMC at all three doses and also by gabapentin [*F* (*5*, 2*2*) = 2*.714*]. A rise in hippocampal dopamine after the development of neuropathic pain was also repressed by both gabapentin and 5,7-DMC at all three doses [*F* (*5*, 2*1*) = 5*.927*]. Moreover, a VCR-induced suppression of hippocampal adenosine was also observed, however, no significant modification was induced by 5,7-DMC, but it was reversed by gabapentin [*F* (*5*, 2*4*) = 3*.475*] *and* [*F* (*5*, 2*3*) = 1*.664*]*.* Whereas the hippocampal level of hypoxanthine was decreased by VCR, it was further decreased by gabapentin, while no significant change was observed after treatment with 5,7-DMC [*F* (*5*, 23) = 1*.523*]. In addition, a VCR-induced upsurge in inosine was decreased by all three doses of 5,7-DMC and gabapentin [*F* (*5*, 21) = 3*.550*] (Table 2) (*one-way ANOVA* with *post hoc* Tukey’s test).

**TABLE 2:** Effect of acute treatment with 5,7-DMC or gabapentin (GBP) on vincristine (VCR) induced changes in hippocampal levels of vitamin C, neurotransmitters and two metabolites of adenosine: One way ANOVA with Tukey’s test was applied. The values (nanogram/mg of wet tissue) are expressed as mean ± SEM, confidence interval = 95% and the significance of differences from the vehicle and VCR group are #, ## and ### and *, ** and *** respectively for *p* < 0.05, *p* < 0.01 and *p* < 0.001.

	Vehicle	VCR	VCR + GBP	VCR +5,7-DMC	VCR +5,7-DMC	VCR +5,7-DMC
	10 mL/kg	0.1 mg/kg	75 mg/kg	30 mg/kg	40 mg/kg	50 mg/kg
**Vitamin C**	1.8 ± 0.17	4.78 ± 0.37	2.04 ± 0.23	2.95 ± 0.18	2.43 ± 0.27	2.72 ± 0.58
**Serotonin**	3.82 ± 1.02	50.2 ± 3.5###	19.93 ± 4.13***	3.42 ± 0.75***	2.56 ± 0.54***	2.52 ± 0.35***
**Norepinephrine**	1.8 ± 0.17	4.78 ± 0.37	2.04 ± 0.23	6.94 ± 1.57	4.17 ± 2.16	4.16 ± 1.46
**Dopamine**	4.96 ± 0.92	69.2 ± 5.8###	30.32 ± 3.46***	13.98 ± 2.14***	9.47 ± 1.6***	8.38 ± 1.94***
**Adenosine**	13.76 ± 1.07	0.33 ± 0.04###	9.3 ± 0.65***	1.56 ± 0.2	1.3 ± 0.08	2.32 ± 0.2
**Inosine**	46.38 ± 8.13	123.6 ± 9.76###	0.34 ± 0.10***	18.2 ± 4.09***	27.1 ± 1.02***	25.66 ± 6.4***
**Hypoxanthine**	28.65 ± 4.28	12.7 ± 2.7##	0.24 ± 0.07*	8.42 ± 1.39	8.42 ± 1.03	11.33 ± 3.27

### 3.7 5,7-DMC reverses vincristine-induced changes in cortical levels of serotonin without affecting other monoamine neurotransmitters

Striatal serotonin was augmented after the development of VINP, and both gabapentin and 5,7-DMC at all doses significantly decreased the elevated serotonin levels [*F* (*5*, 22) = 1*.368*] (*one-way ANOVA* with *post hoc* Tukey’s test). Furthermore, no significant changes in striatal levels of vitamin C, adenosine, dopamine adenosine and hypoxanthine were caused by VCR, gabapentin or 5,7-DMC. Similarly, the level of inosine was not changed by VCR, but it was increased by gabapentin and 5,7-DMC at the two higher doses [*F* (*5*, 21) = 1*.470*] (Table 3).

**TABLE 3:** Effect of acute treatment of 5,7-DMC or gabapentin (GBP) on vincristine (VCR) induced changes in striatal levels of vitamin C, neurotransmitters and two metabolites of adenosine: One way ANOVA with Tukey’s test was applied. The values (nanogram/mg of wet tissue) are expressed as mean ± SEM, confidence interval = 95% and the significance of differences from the vehicle and VCR group are #, ## and ### and *, ** and *** respectively for *p* < 0.05, *p* < 0.01 and *p* < 0.001.

	Vehicle	VCR	VCR + GBP	VCR +5,7-DMC	VCR +5,7-DMC	VCR +5,7-DMC
	10 mL/kg	0.1 mg/kg	75 mg/kg	30 mg/kg	40 mg/kg	50 mg/kg
**Vitamin C**	1.56 ± 0.09	1.07 ± 0.39	2.08 ± 0.24	0.4 ± 0.1	1.35 ± 0.77	1.47 ± 0.42
**Serotonin**	1.28 ± 0.19	13.78 ± 1.18###	2.1 ± 0.52***	1.52 ± 0.51***	2.1 ± 0.53***	1.78 ± 0.14***
**Norepinephrine**	1.88 ± 0.24	1.07 ± 0.39	2.08 ± 0.24	5.2 ± 0.87	4.3 ± 0.77	5.15 ± 0.15
**Dopamine**	13.81 ± 1.57	16.26 ± 2.3	14.52 ± 2.51	13.98 ± 1.96	15.21 ± 1.44	13.99 ± 0.31
**Adenosine**	3.62 ± 0.3	1.76 ± 0.47	2.75 ± 0.42	2.22 ± 0.3	1.15 ± 0.25	2.78 ± 0.8
**Inosine**	15.53 ± 3.05	12.92 ± 0.6	44.4 ± 6.18***	14.78 ± 2.08	26.93 ± 2.98**	46.23 ± 7.3***
**Hypoxanthine**	18.26 ± 1.31	16.74 ± 2.62	20.0 ± 3.71	15.46 ± 2.42	19.08 ± 2.11	17.45 ± 1.67

## 4 Discussion

VINP has been extensively studied, and diverse cellular and molecular targets have been explored for their potential to ameliorate this side effect problem. In this regard, a variety of natural and synthetic compounds, primarily those with antioxidant potential in the attenuation of VINP, have been examined (Khan et al., 2019; Chen et al., 2020; Lee et al., 2020; Starobova et al., 2019; Zhou et al., 2020; Khan et al., 2021; Zhu et al., 2021; Khodaei et al., 2022; Fouda et al., 2023). In our study, VCR administration caused a significant decrease in tail immersion withdrawal latency when assessed on days 10 and 14 of the treatment protocol, and this has also been reported in other studies (Khan et al., 2019; Amirkhanloo et al., 2020; Kim H. et al., 2021). The suggested mechanisms underlying adverse actions of VINP include: swelling of mitochondria in axons intracellularly, leading to the release of calcium ions and apoptosis; increased substance P levels in the spinal cord (Chiba et al., 2022); an involvement of altered adenosine signaling in pain transmission (Zhou et al., 2022); increased release of substances (TNF-α, an adipocytokine, interleukin-1 [IL-1] and IL-6 (cytokines) and NO in glial cells, macrophages, and Langerhans cells); downregulation of IL-10 in the spinal dorsal horn; increased NOS in the spinal cord dorsal horn, increased 5-HT2A receptors in dorsal horn and dorsal root ganglion neurons; increased ROS, which affect serine protease activity and decrease endorphins in the spinal cord as well as the dorsal root ganglion; and increased serine proteinase that inactivates endorphins (Thibault et al., 2008; Addington and Freimer, 2016). Administration of 5,7-DMC reversed the decreased tail withdrawal latency caused by VCR, and this accords with other studies on VCR in combination with other agents (Khan et al., 2019; Akbar et al., 2020; Kim T. Y. et al., 2021), implying that 5,7-DMC has successfully reversed thermal hyperalgesia. The positive control, gabapentin, induced robust effects against thermal hyperalgesia, which is supported by several studies in rodents (Singh et al., 2019; Khan et al., 2021; Khan et al., 2022), suggesting that 5,7-DMC has comparable analgesic effects to gabapentin and might be used for VINP. The reported mechanisms of action of gabapentin in suppressing VINP, include calcium channel inhibition by binding to the α2δ-1 subunit; inhibition of the release of neurotransmitters, including substance-P; glutamate and calcitonin-gene-related-peptide (CGRP); reducing inflammation (by decreasing astrocyte numbers and inhibition of microglial activation); inhibition of NMDA receptors, decreasing protein kinase-C over expression; impeding anterograde neuronal trafficking; decreased trafficking of complexes of β4a-bound Ca_v_2.1 in the plasma membrane; activation of descending pain inhibitory pathways and reducing TRPV1 channel expression (Kukkar et al., 2013; Patel and Dickenson, 2016).

Daily VCR administration caused a decrease in the paw withdrawal threshold on protocol days 10 and 14, and this has been described earlier in mice (Nawaz et al., 2018; Khan et al., 2021; Sahranavard et al., 2022) and rats (Singh et al., 2019; Kim et al., 2020). In relation to this, VINP is associated with an increased expression of cyclooxygenase-2 (Cox-2), TNF-α, interlukin-1β and NF-κB (nuclear factor-kappa B) (Ali et al., 2022) involving Aβ- and C-fibers (Nagi et al., 2011; Ameyaw et al., 2014). Administration of 5,7-DMC reversed the VCR decrease in paw withdrawal threshold, and this action has also been observed in other studies on cisplatin or VCR, co-administered along with other test agents (Akbar et al., 2020; Kim et al., 2020; Lee et al., 2020), suggesting that 5,7-DMC has anti-allodynic effects which may be useful in VINP. Gabapentin as the positive control, caused significant effects on paw withdrawal threshold, which is supported by several studies in rats and mice at different doses (Akbar et al., 2020; Khan et al., 2021; Ali et al., 2022; Basit et al., 2022; Jain et al., 2022). The anti-allodynic effects seen in TLR_4_ −/− mice and following minocycline treatment, provide additional evidence for an immune-driven pathophysiology. TLR4 receptor inhibitors, such as TAK-242, also offer additional therapy options for VIPN; however, these effects need to be examined in upcoming experimental and clinical investigations (Boyette-Davis et al., 2011).

Dynamic allodynia was clearly developed during the VCR administration protocol and this concurs with previous studies (Field et al., 1999; Khan et al., 2019). Subsequently, administration of 5,7-DMC reversed the decreased withdrawal latency induced by subacute VCR dosing, as observed with different agents in earlier studies (Vashistha et al., 2017; Akbar et al., 2020). This substantiates an anti-allodynic effect of 5,7-DMC and as such, it may be a good candidate for managing VINP. Similarly, our positive control, gabapentin, evoked distinct activity against VCR induced dynamic allodynia (Khan et al., 2019). Recombinant human GABA_A_ receptor responses to 6-methylflavanone are positively allosterically modulated, making it a target for studies of clinical diseases involving this kind of activity. Thus, in a model of peripheral neuropathy, 6-methylflavanone lengthened the paw withdrawal latency in the dynamic allodynia test. In order to clarify its mechanisms of action, it was also ascertained that 6-methoxyflavanone has a high antagonistic propensity towards both COX-1 and COX-2 enzymes (Santos et al., 2022). In addition, VCR induced cold allodynia was manifested by a hastened paw response to acetone application in concurrence with earlier reports (Ali et al., 2022; Fouda et al., 2023). 5,7-DMC reversed VCR induced cold allodynia, implying a possible future candidacy of 5,7-DMC against VINP. A comparable finding has been seen with coenzyme Q10 (Elshamy et al., 2022), and gabapentin, caused a similar effect against VCR induced allodynia, which is supported by a previous study (Forouzanfar et al., 2023).

VCR dosing in our investigation increased frontal cortical dopamine concentrations, and a similar outcome has been shown after neuropathic pain develops due to chronic constriction injury (Kang et al., 2023). The concentration of dopamine in the frontal cortex was decreased by 5,7-DMC after VCR treatment, whereas gabapentin caused a further increase in its concentration. This infers that 5,7-DMC is better than gabapentin regarding frontal cortical levels of dopamine, because further disruption, instead of normalization of dopamine, was caused by the positive control, while 5,7-DMC effectively normalized its level. In addition to this, gabapentin possesses the therapeutic drawback of causing dependence (Ferreira et al., 2023), suggesting on two counts, that 5,7-DMC warrants further investigation in VINP.

Increased activation of the serotonergic system in the CCI model of neuropathic pain has been previously demonstrated (Palazzo et al., 2006), which is consistent with the current study in which both gabapentin and 5,7-DMC reversed the increase in serotonin concentration caused by subacute VCR treatment. In another study, 5HT2A knockout mice did not develop neuropathic pain after VCR treatment (Thibault et al., 2008), and the frontal cortex has been reported to play a role in pain processing (Metz et al., 2009). To the best of our knowledge, no study has quantified serotonin levels in the frontal cortex after the development of VINP. Administration of an inhibitor of serotonin synthesis (para-chlorophenylalanine) also increased the analgesic action of *Ocimum sanctum* in the tail flick test (Khanna and Bhatia, 2003), which is consistent with our findings. In this context, our results suggest that 5,7-DMC reverses VINP by normalizing the level of serotonin in the frontal cortex. It has been established that serotonin receptors modulate both sensory and emotional components of pain, and 5-HT3 as well as 5-HT7 receptors have a bimodal role in controlling pain centrally (Neugebauer, 2020; de Kort et al., 2022). Hence, it may be advocated that there is a place for 5,7-DMC in VINP via reversal of disrupted frontal cortical serotonin.

The effect of VINP on the concentration of vitamin C in the frontal cortex has not been studied to date, although VINP was reportedly diminished by the intrathecal administration of L-ascorbic acid (Li G. et al., 2021). Our results showed that the frontal cortical concentration of vitamin C was decreased by VCR, and that 5,7-DMC at a higher dose, reversed that particular disruption, whereas gabapentin did not, further signifying that 5,7-DMC is better than gabapentin in reversing VINP. The plasma concentration of vitamin C has been shown to be lower in patients with postherpetic neuralgia (Chen et al., 2009; Wang et al., 2020), which corroborates the results of our study. What is more, lower vitamin C concentrations in the frontal cortex may give rise to increased reactive oxygen species and hence, increased neuropathic pain. In contrast, vitamin C has neuroprotective properties (Bonnet, 2019) and 5,7-DMC normalizes its level in the frontal cortex suggesting that 5,7-DMC may well reverse VINP via this mechanism.

The effect on noradrenaline concentration in the frontal cortex during VINP has not undergone scrutiny, however, both SSRIs and SNRIs have been demonstrated to attenuate VINP (Katsuyama et al., 2014). We found that the level of noradrenaline was marginally decreased in the frontal cortex after VCR, as opposed to 5,7-DMC treatment at higher doses, which increased it. The concentration of adenosine in the frontal cortex was also decreased after the development of neuropathic pain and this was reversed by both gabapentin and 5,7-DMC. It has already been reported that inhibition of adenosine kinase and the resultant increase in the level of adenosine, significantly relieves neuropathic pain (Little et al., 2015), and this strongly supports our findings. Moreover, activation of A1 receptors has been shown to produce significant effects against VINP (Kim et al., 2020) and likewise, activation of A2A, and A3 receptors also has an inhibitory action against neuropathic pain (Zhou et al., 2022).

This study is the first to describe a decrease in adenosine levels in the frontal cortex after VINP development. Additionally, the level of the adenosine metabolite, hypoxanthine, in the frontal cortex was increased following VCR neuropathy and it was further increased by gabapentin but decreased by 5,7-DMC suggesting an improved pharmacological profile of 5,7-DMC in comparison to gabapentin with reference to normalization of adenosine metabolism. The increase in hypoxanthine level caused by VCR may have originated from increased catabolism of adenosine in the frontal cortex, and this may have been subsequently inhibited by 5,7-DMC. Intriguingly, no significant changes were observed in inosine levels in the current study.

We observed that VCR elevated the dopamine concentration in the hippocampus which was then decreased by 5,7-DMC more effectively than gabapentin, implying that 5,7-DMC is better than the positive control regarding the levels of dopamine in hippocampus. It has been demonstrated that the hippocampus is involved in pain processing, and hippocampal abnormalities have been linked with chronic pain (Mokhtari et al., 2019). Moreover, D1 and D2 dopamine receptor antagonism in the hippocampus can prevent antinociception (Rezaee et al., 2020).

We have shown that gabapentin and 5,7-DMC both reversed the VCR generated hippocampal increase in the concentration of serotonin. In relation to this, decreased levels of serotonin in the hippocampus have been observed in the chronic constriction injury (CCI) model of neuropathic pain (Jiang et al., 2018). This is inconsistent with our present study and may be ascribed to differences in the mechanisms of VCR activity, especially since 5HT2A knockout mice did not develop neuropathic pain following VCR treatment (Thibault et al., 2008). 5-HT3 receptors have been shown to be involved in the analgesic effect of tramadol. In addition, a 5-HT3 receptor antagonist, has been documented to modulate hyperalgesia (Liang et al., 2011) and also inhibit tramadol induced analgesia (Arcioni et al., 2002). In our study, administration of ondansetron but not naloxone, reversed the effects of 5,7-DMC on VINP, implying that there is a role of 5-HT3 receptors in the analgesic effect of 5,7-DMC. In relation to this, some anticancer drugs modify 5-HT3 receptor activity, although VCR has no such effect (Nakamura et al., 2018). 5,7-DMC suppresses the production of IL-1β, TNFα and IL-8 in the colorectal carcinoma HT-29 cell line (Lee et al., 2022). Similarly, it inhibits the expression of IL-8 in the cystic fibrosis bronchial epithelial IB3-1 cell line (Borgatti et al., 2011), and inhibits the expression of IL-4 in RBL-2H3 cells, which are commonly used in immunological experiments, inflammation and allergy (Kim H. et al., 2021). Furthermore, 5,7-DMC decreases the concentration of monoamine oxidase-A, which is responsible for metabolizing monoamines (Yang and Wang, 2018). It is also a potent inhibitor of amyloid beta-40 (Aβ40) aggregation (Kowalczyk et al., 2022), which is known to impair dopaminergic neurotransmission. No study to date has reported the interaction of 5,7-DMC with opioid receptors. However, pretreatment with the opioid receptor antagonist naloxone hydrochloride, significantly reverses the antinociceptive effect of bergamot essential oil which contains 5,7-DMC. Moreover, pretreatment with the peripherally acting *μ*-opioid receptor antagonist naloxone methiodide, effectively reverses bergamot essential oil antinociception (Sakurada et al., 2011). 5-HT3 receptors also mediate formalin-induced hyperalgesia and allodynia (Bravo-Hernández et al., 2012) but to the best of our knowledge, there is no data available on the evaluation of 5-HT levels in the hippocampus after the development of VINP. Correspondingly, because ondansetron blocked the effect of 5,7-DMC on VINP, this convincingly suggests that 5,7-DMC targets 5-HT3 receptors in the serotonergic system in reversing VINP.

Equally, the effect of VINP on the concentration of vitamin C in the hippocampus has not yet been studied. Our results showed that the concentration of vitamin C was not significantly increased by VCR in the hippocampus and neither 5,7-DMC nor gabapentin modified the lack of VCR’s activity in this respect.

In the striatum, VCR did not significantly increase the dopamine concentration and it was not modified by 5,7-DMC or gabapentin. It has been demonstrated that the striatum is involved in pain processing, and abnormalities in the striatum are associated with chronic pain (Gemikonakli et al., 2019), however, the experimental outcomes we obtained would suggest that there is little or no dopaminergic involvement in VINP or its reversal by 5,7-DMC.

The current increase we observed in the plasma level of TNF-α caused by VCR was significantly decreased by gabapentin as well as 5,7-DMC at all three doses employed. Enhanced TNF-α concentrations after the development of VINP have previously been described (Zhou et al., 2018), and this is in agreement with our findings which also suggest that TNF-α is associated with the anti-VINP activity of 5,7-DMC.

## 5 Conclusion

5,7-DMC attenuates VINP and is a potential candidate for further studies. It acts by targeting 5-HT3 receptors, but not the opioidergic system to attenuate VINP and 5,7-DMC at all three doses significantly lowered plasma TNF-α levels. Our results are the first to describe changes in the levels of neurotransmitters in the frontal cortex, striatum, and hippocampus after VINP development. Interestingly, in reversing the changes in the levels of some monoamine neurotransmitters in the brain caused by VCR, 5,7-DMC showed stronger effects than gabapentin which was utilized as a positive control. It was deduced that, there is a potential role of 5-HT3 receptors and monoamines in the amelioration of VINP induced by 5,7-DMC, and the use of this compound warrants further investigation.

## 6 Limitations of the study

More specific receptor studies, especially with 5-HT3 knock-out animals, in addition to an evaluation of possible activity of 5,7-DMC in modifying other inflammatory mediators at higher 5,7-DMC doses would have been beneficial. Furthermore, 5,7-DMC could be screened at other targets or receptors for treating VINP. Addressing these limitations is warranted in further investigative studies for the future.

## Data Availability

The original contributions presented in the study are included in the article/Supplementary material, further inquiries can be directed to the corresponding author.
